# Regulation of Multidrug Resistance Proteins by Genistein in a Hepatocarcinoma Cell Line: Impact on Sorafenib Cytotoxicity

**DOI:** 10.1371/journal.pone.0119502

**Published:** 2015-03-17

**Authors:** Juan Pablo Rigalli, Nadia Ciriaci, Agostina Arias, María Paula Ceballos, Silvina Stella Maris Villanueva, Marcelo Gabriel Luquita, Aldo Domingo Mottino, Carolina Inés Ghanem, Viviana Alicia Catania, María Laura Ruiz

**Affiliations:** 1 Institute of Experimental Physiology (IFISE-CONICET), Faculty of Biochemical and Pharmaceutical Science, Rosario National University, Rosario, Argentina; 2 Institute of Pharmacological Investigations (ININFA-CONICET), Faculty of Pharmacy and Biochemistry, University of Buenos Aires, Buenos Aires, Argentina; University of Navarra School of Medicine and Center for Applied Medical Research (CIMA), SPAIN

## Abstract

Hepatocellular carcinoma (HCC) is the fifth most frequent cancer worldwide. Sorafenib is the only drug available that improves the overall survival of HCC patients. P-glycoprotein (P-gp), Multidrug resistance-associated proteins 2 and 3 (MRP2 and 3) and Breast cancer resistance protein (BCRP) are efflux pumps that play a key role in cancer chemoresistance. Their modulation by dietary compounds may affect the intracellular accumulation and therapeutic efficacy of drugs that are substrates of these transporters. Genistein (GNT) is a phytoestrogen abundant in soybean that exerts its genomic effects through Estrogen-Receptors and Pregnane-X-Receptor (PXR), which are involved in the regulation of the above-mentioned transporters. We evaluated the effect of GNT on the expression and activity of P-gp, MRP2, MRP3 and BCRP in HCC-derived HepG2 cells. GNT (at 1.0 and 10 μM) increased P-gp and MRP2 protein expression and activity, correlating well with an increased resistance to sorafenib cytotoxicity as detected by the methylthiazole tetrazolium (MTT) assay. GNT induced P-gp and MRP2 mRNA expression at 10 but not at 1.0 μM concentration suggesting a different pattern of regulation depending on the concentration. Induction of both transporters by 1.0 μM GNT was prevented by cycloheximide, suggesting translational regulation. Downregulation of expression of the miR-379 by GNT could be associated with translational regulation of MRP2. Silencing of PXR abolished P-gp induction by GNT (at 1.0 and 10 μM) and MRP2 induction by GNT (only at 10 μM), suggesting partial mediation of GNT effects by PXR. Taken together, the data suggest the possibility of nutrient-drug interactions leading to enhanced chemoresistance in HCC when GNT is ingested with soy rich diets or dietary supplements.

## Introduction

Hepatocellular carcinoma (HCC) is the most common type of primary liver cancer. It ranks as the fifth most frequent cancer and the third leading cause of cancer mortality worldwide [1]. The traditional management of HCC depends on the stage at which the disease is diagnosed and consists of surgical resection, ablation or liver transplantation [2]. Conventional chemotherapy with agents such as 5-fluorouracil, epirubicin and doxorubicin has been applied as palliative treatment but survival improvement in the majority of the patients is negligible [3]. Sorafenib (Sfb) is a small tyrosine kinase inhibitor that has been proved to be successful in renal cell carcinoma treatment. It targets vascular endothelial growth factor receptors, Raf kinase and platelet-derived growth factor receptor-b, thus inhibiting tumor proliferation and angiogenesis [3, 4]. A large prospective randomized controlled trial done in 2008 showed that Sfb treatment improved overall survival in advanced stages of HCC [5]. This was further confirmed in a second trial done on Asian population [6].

A major mechanism by which many cancer cells develop resistance to chemotherapy is known as multidrug resistance, a phenotype characterized by diminished intracellular drug accumulation leading to treatment failure [7]. Multidrug resistance has been correlated to overexpression of transporters belonging to the ATP binding cassette (ABC) superfamily like P-gp/ABCB1, MRP2/ABCC2, MRP3/ABCC3 and BCRP/ABCG2 among other factors [7–9]. Physiologically they localize to the plasma membrane of several epithelial cells such as hepatocytes, renal tubular cells and enterocytes [7–10]. P-gp transports mainly amphipathic, hydrophobic and cationic compounds [7, 10]. Its overexpression has been associated with increased resistance to doxorubicin in HCC [11, 12]. MRP2 and MRP3 transport organic anions usually conjugated with glutathione, sulfate or glucuronic acid [8, 9]. In a study involving HCC patients who underwent tumor resection, increased MRP2 expression was associated with a poorer response to neoadjuvant chemotherapy with cisplatin [13]. In other malignancies, like esophageal squamous cell carcinoma, MRP2 was shown to regulate the sensitivity to the treatment with 5-fluorouracil, doxorubicin and cisplatin and, moreover, its overexpression was correlated with a poorer prognosis of the disease [7–10]. MRP3 was associated with resistance to methotrexate [14]. BCRP transports a wide range of conjugated and unconjugated xenobiotics and was demonstrated to mediate resistance to chemotherapeutic agents like daunorubicin and mitoxantrone [15, 16]. As far as HCC is concerned, there is evidence associating BCRP overexpression with resistance to doxorubicin *in vitro* [17]. It was reported that Sfb intracellular accumulation is modulated by P-gp, MRP2 and BCRP [18, 19] due to the extrusion of either Sfb itself or its metabolites, which exhibit also chemical properties typical of P-gp and MRP2 substrates [10, 20, 21]. Consequently, acquired resistance to Sfb due to an increased drug extrusion (e.g. after transporter induction) may be expected.

The expression and activity of these transporters can be modulated by factors like diet, hormones, aging, diseases or drugs [10, 22, 23]. Regulation can occur either at the transcriptional or posttranscriptional level, resulting in changes in mRNA levels and, usually, in protein content, or at translational or posttranslational level resulting in changes in protein content and dissociation from mRNA levels [9, 10, 22, 24]. Induction of transporter expression by xenobiotics is usually mediated by nuclear receptors. Pregnane X receptor (PXR, NR1I2) is a nuclear receptor considered the main xenosensor regulating genes involved in drug disposition, including P-gp, MRP2, MRP3 and BCRP [9, 22, 25]. Recently, it was demonstrated that miRNAs are additional factors involved in modulating transporter expression. For instance, miR-223 and miR-379 were shown to affect the expression of P-gp and MRP2, respectively [12, 26].

Genistein (GNT), a phytoestrogen that belongs to the family of isoflavones, is ingested as a component of vegetables (e.g. soy beans, fava beans, lupins) or as a dietary supplement [27, 28]. GNT is considered to exert antioxidant and anticarcinogenic effects [29, 30]. However, it is controversial whether these beneficial properties are achieved at plasma concentrations compatible with GNT ingestion as part of a soy rich diet or GNT containing supplements, which range between 0.5 and 10 μM [30, 31, 32]. Up to date there is little information on GNT potential to act as modulator of the expression and activity of ABC transporters in HCC. If so, a reduced intracellular accumulation of chemotherapeutic agents, substrates of these transporters, would be expected. This fact may account for a reduced therapeutic efficacy. The aim of this work was to evaluate the effect of GNT on the expression and activity of P-gp, MRP2, MRP3 and BCRP in the human HepG2 cell line, derived from HCC, and the underlying molecular mechanisms. Additionally, whether the observed changes in transporter activities modify the Sfb cytotoxic effect was also assessed.

## Materials and Methods

### Chemicals

1-chloro-2,4-dinitrobenzene (CDNB), cycloheximide, dinitrophenyl-S-glutathione (DNP-SG), genistein (GNT), MK571, 3-(4,5-dimethylthiazol-2-yl)-2,5-diphenyltretazolium bromide (methylthiazole tetrazolium, MTT), perchloric acid, phenylmethylsulfonyl fluoride and leupeptin were from Sigma-Aldrich (St. Louis, MO, USA). PSC833 and calcein-AM were from Santa Cruz Biotechnology (Santa Cruz, CA, USA). Sorafenib (Sfb) was from Cayman Chemicals (Ann Arbor, MI, USA). DMSO was purchased from Merck (Darmstadt, HE, Germany). All other chemicals were of analytical grade purity.

### Cell culture and treatments

As reported for HCC [33, 34], HepG2 cells present reduced expression of ERα and absence of ERβ [35]. Nevertheless, they retain cell polarity, this being essential for proper localization of membrane transporters [36]. HepG2 cells also conserve the expression of PXR [37].

HepG2 cells were obtained from the American Tissue Culture Collection (Rockville, MD, USA). Cells were grown in Dulbecco's modified Eagle's medium (DMEM) and Ham's F-12 medium (Invitrogen, Carlsbad, CA, USA) at a 1:1 proportion, supplemented with 10% FBS (PAA, Pasching, Austria), 2 mM L-glutamine, a mixture of antibiotics (5 mg/ml penicillin, 5 mg/ml streptomycin and 10 mg/ml neomycin) and 0.1 mg% insulin (Invitrogen). Cells were incubated at 37°C in a humidified atmosphere containing 5% CO_2_ as described [35]. For the treatments, unless otherwise stated, cells were seeded in 6-well plates at a density of 3.5x10^5^ cells/well and cultured for 24 h. Following, GNT was dissolved in DMSO and added to treatment medium at different concentrations (0.1, 1.0 or 10 μM) within the range reported in plasma for individuals consuming a soy-rich diet or GNT containing supplements [31, 32]. Only DMSO was added to control cells (C). The final concentration of DMSO in the culture media was always below 0.1%. Cells were cultured in the presence of GNT or DMSO for 48 h. Treatment medium consisted of the growth medium described above with FBS being replaced by Charcoal-dextran stripped FBS (Hyclone, Logan, UT, USA), which provides a lower level of steroid hormones than standard FBS [35]. All treatments were performed in phenol red-free culture medium. To assess whether the effect of GNT (1.0 μM) treatment on P-gp and MRP2 occurs at the translational level, cells were coincubated with cycloheximide (100 μM) as a protein synthesis inhibitor [38].

### Western blot and real time RT-PCR studies

The effect of GNT on P-gp, MRP2, MRP3 and BCRP protein expression was initially assessed in cell lysates. HepG2 cells were washed twice with cold PBS and scraped with RIPA buffer (Thermo Scientific, Rockford, IL, USA) supplemented with phenylmethylsulfonyl fluoride (17 μg/ml) and leupeptin (15 μg/μl) as protease inhibitors. Lysates were passed 20 times through a 25G needle and subjected to protein concentration assessment [37].

To evaluate more specifically the expression of P-gp and MRP2 at the cell surface, plasma membranes from HepG2 cells were isolated as described by Kubitz et al. [39]. Briefly, the cells were scraped in a buffer containing Tris 20 mM, sucrose 250 mM, EGTA 5 mM and MgCl_2_ 1 mM supplemented with protease inhibitors. Cell lysis was achieved passing the cell suspension 20 times through a 25G needle. Plasma membranes were obtained after centrifugation (5 min, 1000 g, 4°C). Protein concentration was quantified by the Lowry method [37].

Western blotting and bands quantification were performed as previously described [35]. Primary antibodies were: anti-BCRP, BXP-21; anti-GAPDH, FL-335; anti-P-gp, H-241 (Santa Cruz Biotechnology); anti-MRP2, M_2_III-6 (Enzo Life Sciences, Farmingdale, NY, USA); anti-MRP3, M_3_II-21 and anti-β-actin, A-2228 (Sigma-Aldrich). HRP-conjugated goat anti-Mouse IgG (H+L) (Thermo Scientific) was used as secondary antibody for BCRP, MRP2 and β-actin detection, whereas a HRP-conjugated donkey anti-Rabbit IgG (H+L) (Thermo Scientific) was used as secondary antibody for GAPDH, P-gp and MRP3 detection.

Real time RT-PCR was performed only if changes in protein expression were detected by western blotting. Total RNA was isolated using TRIzol reagent (Invitrogen). cDNA was synthesized from 1 μg of total RNA with the Superscript III Reverse Transcriptase (Invitrogen) using random hexamers according to manufacturer's instructions. Real time PCR reactions were carried out on a MX3000P system (Agilent Technologies, Santa Clara, CA, USA) with Platinum Taq DNA Polymerase (Invitrogen). The amount of template was quantified with SYBR Green (Invitrogen). Primers were used at a final concentration of 1 μM. Primer sequences were: MDR1(F): 5'CCAAAGACAACAGCTGAAA3'; MDR1(R): 5'TACTTGGTGGCACATAAAC3'; MRP2(F): 5'AGGTTTGCCAGTTATCCGTG3'; MRP2(R): 5'AACAAAGCCAACAGTGTCCC3'; 18S(F): 5'CGCCGCTAGAGGTGAAATTC3'; 18(R): 5'TTGGCAAATGCTTTCGCT3' [40]. The thermocycling regime was 95°C for 2 min followed by 40 cycles of 95°C for 15 sec, 55°C for 30 sec and 72°C for 30 sec. Relative levels of MDR1 (encoding P-gp) and MRP2 mRNA normalized to 18S rRNA were calculated based on the 2^-ΔΔCt^ method [35]. Specificity of each reaction was verified by the dissociation curve between 55°C and 95°C.

### Assessment of transport activities

Activity measurements were performed in those GNT groups exhibiting increases in transporter expression. The activity of P-gp was assessed measuring intracellular retention of fluorescent calcein, which inversely correlates with transporter activity [41]. Cells were cultured in 6-well plates as previously described, trypsinized, resuspended, and incubated (15 min, 37°C) in 500 μL of growth medium containing 0.5 μM of calcein-AM. They were then washed and resuspended in cold PBS. Intracellular fluorescence was quantified in a Cell Sorter BD FACSAria II device (BD Biosciences, San Jose, CA, USA) using a blue laser (488 nm, 20 mW) and FITC detection filter (530/30 nm). Measurements were restricted to live cells by gating them according to the typical forward and side scatter pattern of live cells. P-gp participation was confirmed using the specific inhibitor PSC833 (10 μM) [41].

The activity of MRP2 was quantified as previously reported through determination of the amount of the model substrate dinitrophenyl-S-glutathione (DNP-SG) extruded into the incubation medium [40]. Briefly, cells were cultured in 6-well plates and treated with GNT (1.0 or 10 μM, 48 h) as described above. Then, treatment medium was replaced with fresh medium containing CDNB (0.5 mM) and cells were incubated at 10°C for 30 min to allow CDNB to passively diffuse into the cytosol. In this condition, CDNB conversion to DNP-SG occurs spontaneously, i.e independently of GST activity [40]. At the end of incubation the medium was rinsed and cells were promptly washed twice with cold PBS. To evaluate the rate of DNP-SG secretion, cells were incubated with HBSS buffer at 37°C for 60 min. At the end of incubations, aliquots were subjected to centrifugation (3 min, 300 g, 4°C), supernatants were treated with 10% perchloric acid, centrifuged (2 min, 14000 g, 4°C) and the supernatants used for DNP-SG detection by HPLC using a Waters 600 device (Waters, Milford, MA, USA) as described [40]. MRP2 participation was confirmed using MRPs inhibitor MK571 (10 μM) [42].

### Cytotoxicity assays

To further assess the functional consequences of transporter induction we studied the protective role of GNT treatment against the cytotoxicity exerted by sorafenib (Sfb), substrate of P-gp, MRP2 and BCRP [4, 18, 19]. For this purpose HepG2 cells were seeded in 96-well plates (1.5x10^4^ cells/well). After 24 h of culture, fresh medium containing 1.0 or 10 μM GNT was added and cells were further incubated for 48 h. Cells were then rinsed and incubated with different concentrations of Sfb (0–200 μM) for 16 h. P-gp and MRP2 participation was confirmed coincubating the cells either with 10 μM PSC833 (P-gp inhibitor), with 10 μM MK571 (MRP2 inhibitor) or with PSC833 and MK571 (10 μM each). CYP3A4 participation in the modulation of Sfb cytotoxicity was assessed coincubating the cells with 1.0 μM ketoconazole (CYP3A4 inhibitor) [43]. Cell viability was then evaluated through the MTT assay as described [37].

### MicroRNA quantification

Total RNA was isolated with TRIzol (Invitrogen) as described above from control and 1.0 μM GNT treated cells, and the expression of mature miR-223, miR-379 and miR-451 was quantified through Stem Loop RT-PCR followed by quantitative PCR [44]. Stem Loop RT-PCR was performed using 1 μg of total RNA. Stem Loop (SL) oligonucleotides and housekeeping (5S rRNA for miR-223 and U6 snRNA for miR-379 and miR-451) specific primers were used at 50 nM and 750 nM, respectively. Retrotranscription was performed using SuperScript III Reverse Transcriptase (Invitrogen) with specific primers according to manufacturer's instructions. The thermal profile consisted of a first incubation for 30 min at 16°C, followed by 30 min at 42°C, 60 min at 50°C and finally 15 min at 70°C. Primer sequences were: miR-223 SL: 5'GTCTCCTCTGGTGCAGGGTCCGAGGTATTCGCACCAGAGGAGACTGGGGT3' (miRBase accession MIMAT0000280), miR-379 SL: 5'GTCTCCTCTGGTGCAGGGTCCGAGGTATTCGCACCAGAGGAGACCCTACG3' (miRBase accession MIMAT0000733); miR-451 SL: 5' GTCTCCTCTGGTGCAGGGTCCGAGGTATTCGCACCAGAGGAGACAACTCA3' (miRBase accession MIMAT0001631); U6 snRNA: 5'CCAGTGCAGGGTCCGAGGT3'; 5S rRNA: 5'GCGGTCTCCCATCCAAGTAC3'. All stem loop primers were designed to amplify only the specified mature miRNAs yielding a cDNA with the necessary length to be detected in the following real time PCR assays [44].

Real time PCR reactions for microRNA quantification were carried out on a MX3000P system (Agilent Technologies) with Platinum Taq DNA Polymerase and SYBR Green quantification (Invitrogen). Primers were used at a final concentration of 1 μM. cDNA samples were used at 1/50 dilution for miR-223 and 1/10 dilution for miR-379 and miR-451. Primer sequences were: miR-223 (F): 5'GCAGCCTGTCAGTTTGTCA3'; miR-379(F): 5'CGGCCTGGTAGACTATGGA3'; miR-451(F): 5'GCTCGGAAACCGTTACCATTA3'; universal reverse primer: 5'GAGGTATTCGCACCAGAGGA3'; U6 snRNA(F): 5'TGCGGGTGCTCGCTTCGGCAGC3'; U6 snRNA (R): 5'CCAGTGCAGGGTCCGAGGT3'; 5S rRNA (F): 5'TACGGCCATACCACCCTGAA3' and 5S rRNA (R): 5'GCGGTCTCCCATCCAAGTAC3'. The thermocycling regime was 95°C for 1 min followed by 40 cycles of 95°C for 15 sec, 54°C (for miR-223) or 60°C (for miR-379 or miR-451) for 30 sec and 72°C for 40 sec. Relative levels of miR-223 normalized to 5S rRNA and miR-379 or miR-451 normalized to U6 snRNA in control and GNT 1.0 μM treated cells were calculated based on the 2^-ΔΔCt^ method [35]. Specificity of each reaction was verified by the dissociation curve between 55°C and 95°C.

### Knock down of PXR

HepG2 cells (5x10^4^ cells/well) were seeded in 24-well plates, incubated at 37°C and subjected to transfection 24 h later. Human PXR was transiently knocked down with 100 nM of PXR siRNA (h) (Santa Cruz Biotechnology, sc-44057) targeting the human PXR mRNA (PXR^-^ cells). Control siRNA-A (Santa Cruz Biotechnology, sc-37007), a non-targeting siRNA, was used as control (PXR^+^ cells). Transfections were performed using Dharmafect4 Transfection Reagent (Dharmacon, Lafayette, CO, USA) as described [37]. Twenty four h after initiation of transfection, GNT (at 1.0 or 10 μM final concentration) was added to the transfection medium. Twenty four h later, the medium containing siRNA (or control siRNA-A) was removed, fresh treatment medium containing GNT (or vehicle) was added, and cells were further incubated for 24 h. At the end of the incubation, they were rinsed, scraped and used in western blot studies as described above.

### Statistical analysis

Data are presented as mean ± S.D. Statistical analysis was performed using the Student *t* test (for two experimental groups) or One-Way ANOVA followed by Newman-Keuls post hoc test (for more than two experimental groups). Significance was set at p < 0.05. Studies were performed using the GraphPad Prism 3.0 software (GraphPad Software, La Jolla, CA, USA).

In the case of cytotoxicity studies, cell viability measures (% of viable cells) were plotted as a function of the decimal logarithm of Sfb concentration. The curves were best adjusted to a sigmoid curve using the GraphPad Prism 3.0 software (GraphPad Software, La Jolla, CA, USA). The goodness of adjustments was confirmed with R^2^ values, which were always above 0.85.

## Results

### Effect of GNT on protein expression and activity of drug transporters

**Fig 1:**
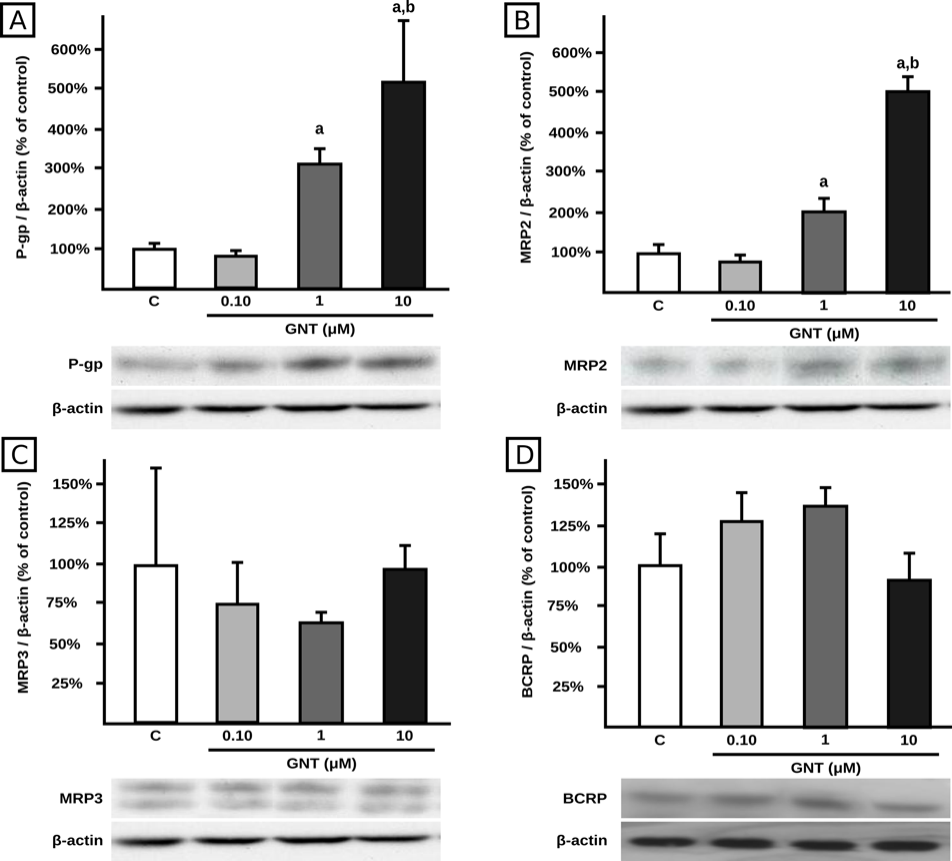
Effect of GNT on transporter expression in cell lysates. Expression of P-gp (panel A), MRP2 (panel B), MRP3 (panel C) and BCRP (panel D) was quantified by western blotting in HepG2 lysates after 48 h of treatment with GNT (0.1, 1.0, or 10 μM) or vehicle (C). Fifteen μg of total protein were loaded in the gels. Transporter O.D. were normalized to β-actin O.D. Uniformity of loading and transfer from gel to PVDF membrane was also controlled through Ponceau S staining. The data (mean ± S.D., n = 3) are expressed as percentage of the normalized transporter expression in control (C) cells. Typical western blot detections are shown at the bottom. a: different from control, b: different from GNT 1.0 μM. p<0.05.

GNT increased protein levels of P-gp and MRP2 at 1.0 μM (+206% and +102%, respectively) and at 10 μM (+402% and +390%, respectively) in cell lysates, with no changes at 0.1 μM concentration, clearly showing a concentration-dependent response (see Fig. 1A and 1B). In contrast, GNT did not affect protein levels of MRP3 (Fig. 1C) or BCRP (Fig. 1D) at any of the concentrations tested.

**Fig 2:**
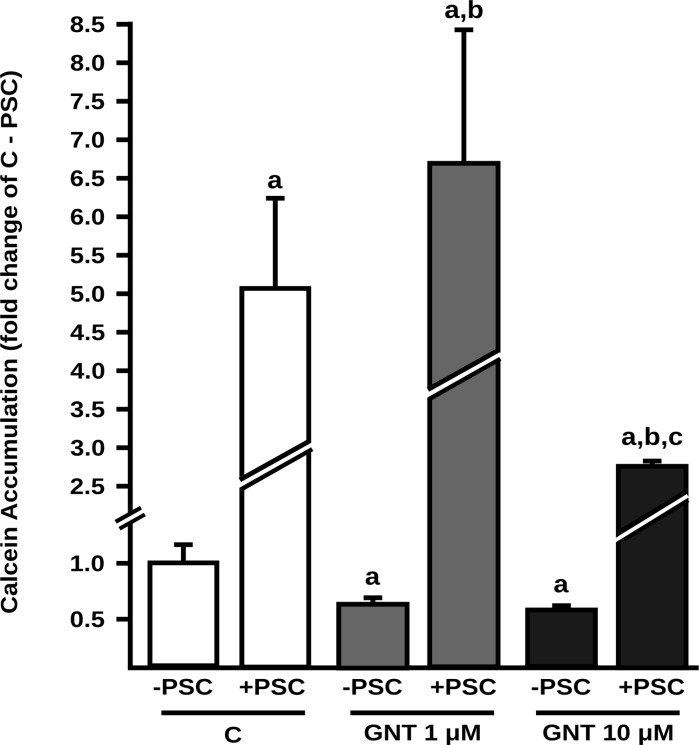
Effect of GNT on P-gp activity. Calcein accumulation is directly associated to accumulation of its non fluorescent precursor calcein-AM (P-gp substrate) and was quantified by flow cytometry in control (C) and GNT treated cells (1.0 and 10 μM, 48 h) in the absence (- PSC) or presence (+ PSC) of the P-gp specific inhibitor PSC833 (10 μM). Data (mean ± S.D., n = 3) are presented as fold change of the calcein accumulation in C—PSC, considered as 1. a: different from C—PSC, b: different from GNT 1.0 μM—PSC; c: different from GNT 10—PSC. p<0.05.

**Fig 3:**
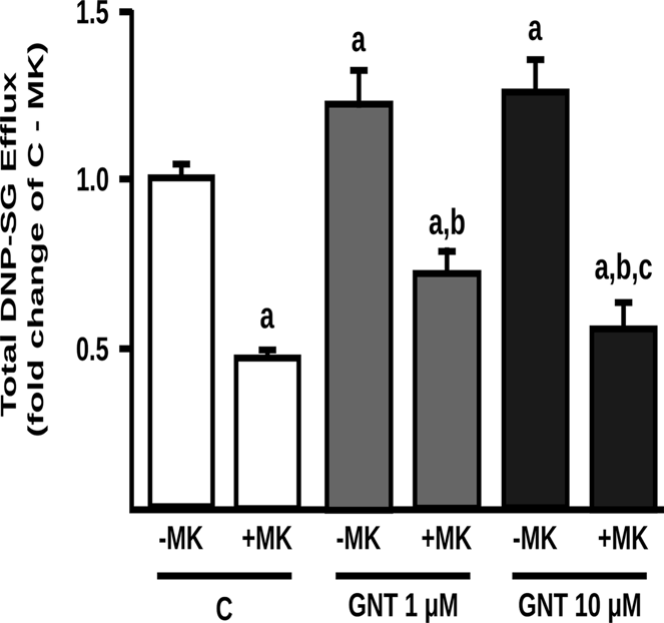
Effect of GNT on MRP2 activity. Extrusion of the MRP2 model substrate DNP-SG was quantified in supernatants of control (C) and GNT treated cells (1.0 and 10 μM, 48 h) in the absence (- MK) or presence (+ MK) of the MRP inhibitor MK571 (10 μM). Data (mean ± S.D., n = 3) are presented as fold change of the DNP-SG extrusion in C—MK, considered as 1. a: different from C—MK; b: different from GNT 1.0 μM—MK; c: different from GNT 10 μM—MK. p<0.05.

To determine the functional impact of P-gp and MRP2 upregulation by GNT, we evaluated their transport activities using two different experimental strategies that were found to be optimal in each case. Intracellular accumulation of calcein was lower in GNT treated cells than in control cells (-37% and -43%, for GNT 1.0 and 10 μM, respectively) (Fig. 2). Intracellular content of calcein was increased by the P-gp specific inhibitor PSC833 in both control and GNT treated cells, confirming the contribution of P-gp to calcein efflux. Also, we observed a higher excretion rate of DNP-SG in 1.0 and 10 μM GNT treated cells (23% and 26%, respectively) (Fig. 3). The addition of MK571 inhibited the efflux of DNP-SG both in control and GNT treated cells, consistent with participation of a MRP transporter.

**Fig 4:**
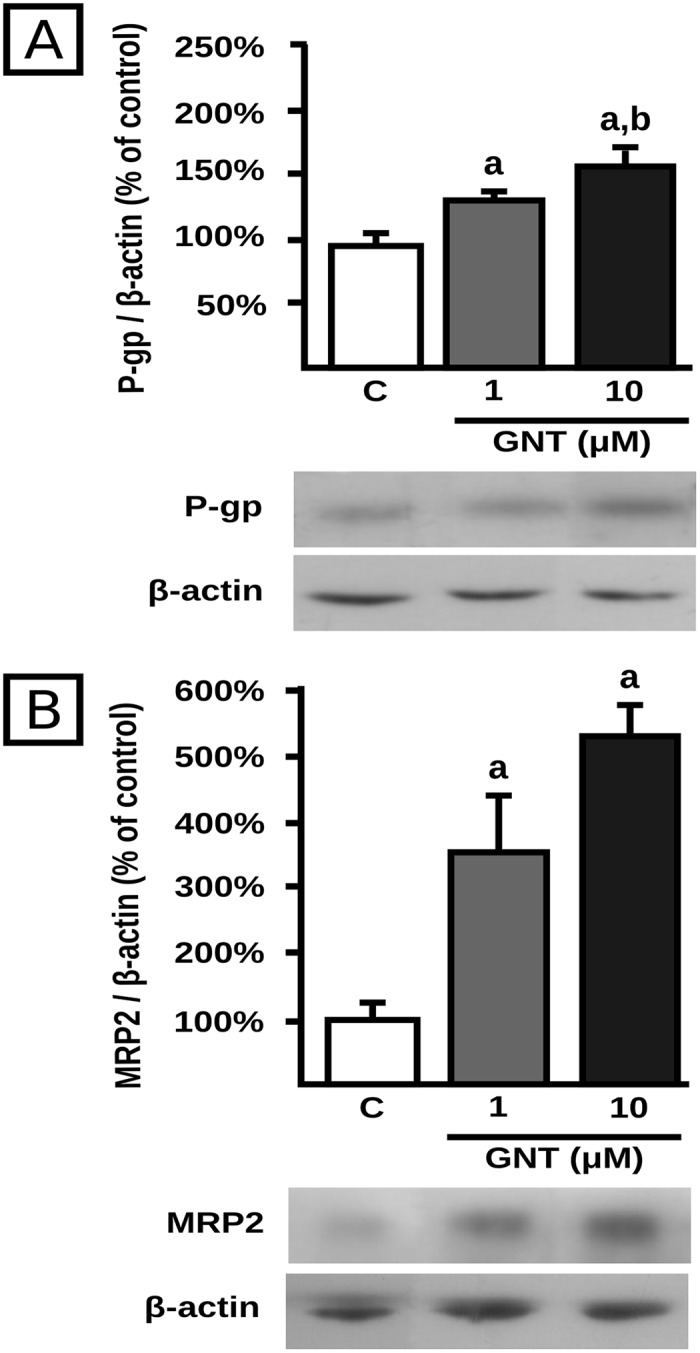
Effect of GNT on transporter expression in crude plasma membranes. Expression of P-gp (panel A) and MRP2 (panel B) was quantified by western blotting in HepG2 crude plasma membranes after 48 h of treatment with GNT (0.1, 1.0, or 10 μM) or vehicle (C). Fifteen μg of crude plasma membrane protein were loaded in the gels. Transporter O.D. were normalized to β-actin O.D. Uniformity of loading and transfer from gel to PVDF membrane was also controlled through Ponceau S staining. The data (mean ± S.D., n = 3) are expressed as percentage of the normalized transporter expression in control (C) cells. Typical western blot detections are shown at the bottom. a: different from control, b: different from GNT 1.0 μM. p<0.05.

Despite the higher P-gp and MRP2 protein expression in lysates from cells incubated with 10 μM of GNT than from those incubated with the 1.0 μM concentration (Fig. 1A and B), the enhancements of transport activities were of similar extent between the two concentrations (Figs. 2 and 3). In an attempt to elucidate the reasons for such discrepancy, we evaluated the effect of GNT (1.0 and 10 μM, 48 h) on P-gp and MRP2 protein expression in crude plasma membranes, since the correct localization of these proteins is essential for their proper function, among other factors [45]. MRP2 protein expression did not exhibit a significant difference between 1 and 10 μM (Fig. 4). Thus, this could be the reason for the unchanged DNP-SG excretion between 1 and 10 μM. However, this may not be the case for P-gp since there is a slight but significant increase in P-gp protein expression in plasma membrane at 10 μM compared with 1.0 μM (Fig. 4), whereas the intracellular levels of calcein were similar between both GNT concentrations.

### Impact of drug transporters induction by GNT on Sfb cytotoxicity

It was of interest to study if GNT pretreatment of HepG2 cells could affect the cytotoxicity produced by Sfb, a drug used for HCC treatment. The protective effect of GNT against Sfb cytotoxicity was evaluated through determination of cell survival. IC_50_ values from control and GNT pretreated cells are shown in Table 1. The results showed that the IC_50_ was higher in cells treated with GNT (1.0 and 10 μM) than in control cells. These effects were prevented by the inhibition of P-gp with PSC833, the inhibition of MRP2 by MK571 and the simultaneous inhibition of both transporters. Thus, an association can be established between upregulation of P-gp and MRP2 and resistance to cytotoxicity exerted by Sfb in HepG2 cells. Additionally, when P-gp was inhibited by PSC833 the cytotoxic effect of Sfb was higher than in the presence of the MRP2 inhibitor, MK571, suggesting a major role of P-gp in cytoprotection, in all groups.

**Table 1:** Effect of GNT on sorafenib-induced cytotoxicity.

	Sfb IC_50_ (μM)		
	Control	GNT 1.0 μM	GNT 10 μM
Without inhibitor	34.8 ± 3.3	46.0 ± 6.9*	43.3 ± 2.8*
PSC833	6.7 ± 2.5^a^	6.0 ± 1.1^a^	8.5 ± 4.3^a^
MK571	18.3 ± 6.0^a^ ^,^ ^b^	25.1 ± 6.3^a^ ^,^ ^b^	17.1 ± 1.1^a^ ^,^ ^b^
PSC833 + MK571	10.9 ± 4.2^a^ ^,^ ^c^	8.5 ± 1.7 ^a^ ^,^ ^c^	8.0 ± 1.2^a^ ^,^ ^c^

IC_50_ values (mean ± S.D., n = 4) were obtained through nonlinear regression.

* different from control (C), p<0.05.

IC_50_ values were also compared within each column.

^a^: different from without inhibitor;

^b^: different from PSC833;

^c^: different from MK571, p<0.05.

### Molecular basis of drug transporters induction by GNT

**Fig 5:**
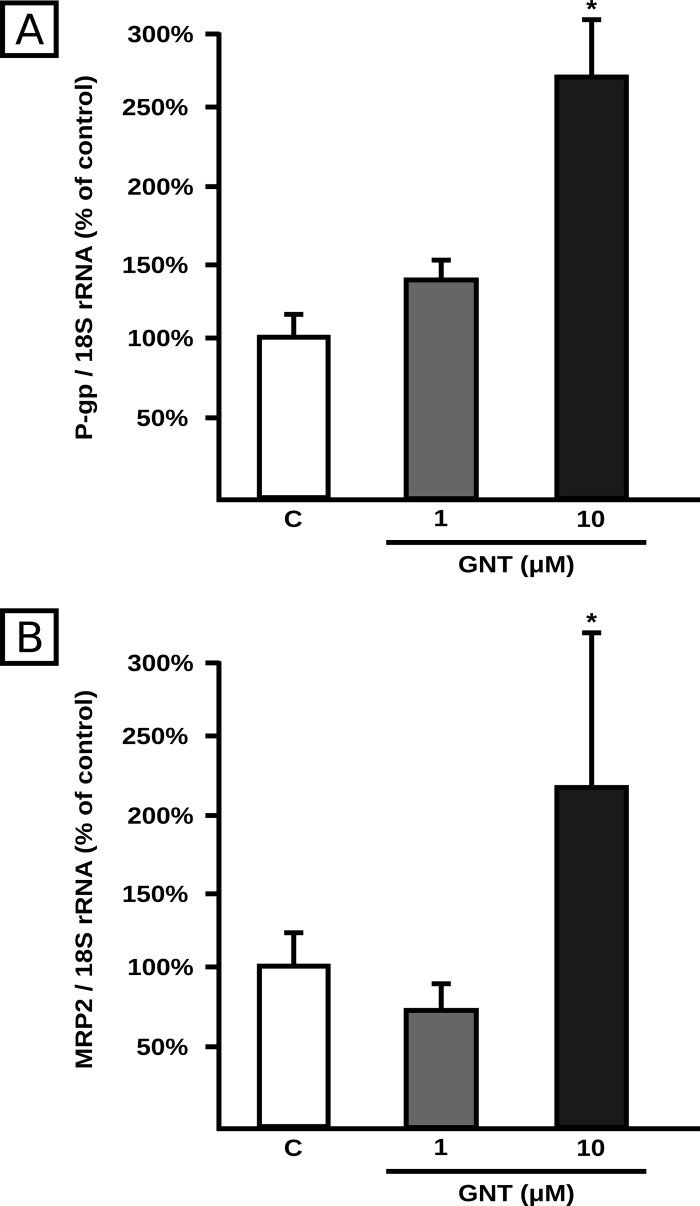
Effect of GNT on transporter mRNA expression. P-gp (panel A) and MRP2 (panel B) mRNA levels were measured by real time RT-PCR using total RNA isolated from HepG2 cells after 48 h of treatment with GNT (0.1, 1.0, or 10 μM) or vehicle (C). Expression of the target genes was normalized to 18S rRNA expression. The data (mean ± S.D., n = 4) are expressed as percentage of the normalized transporter expression in control (C) cells. *: different from all the other groups, p<0.05.

**Fig 6:**
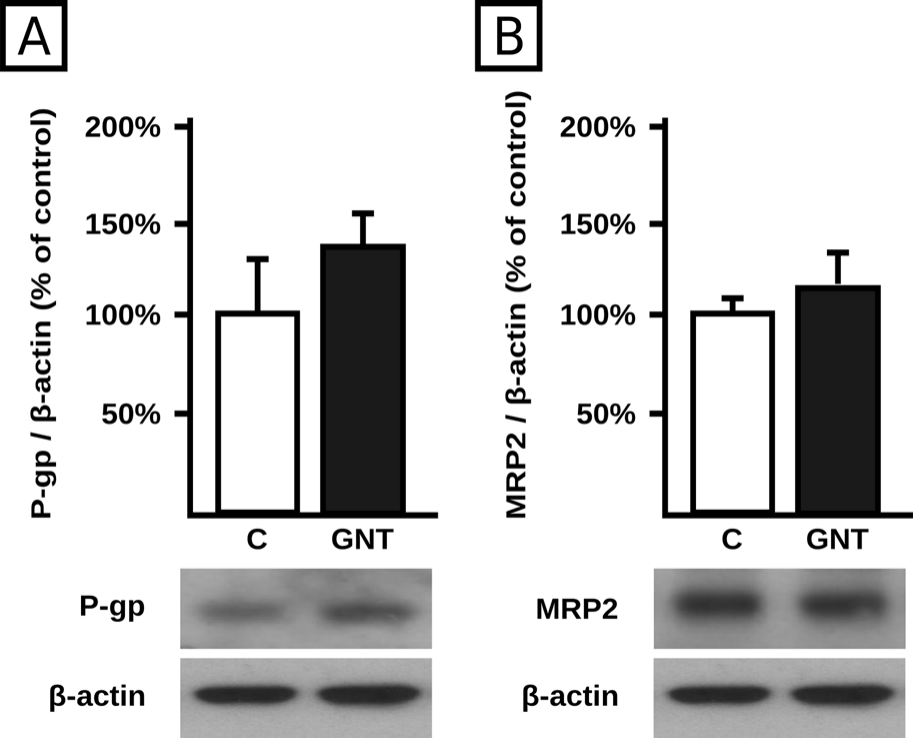
Effect of cycloheximide on transporter induction by GNT. Expression of P-gp (panel A) and MRP2 (panel B) was quantified by western blotting in HepG2 cell lysates after treatment with GNT (1.0 μM, 48 h) or vehicle (C) in the presence of the translation inhibitor cycloheximide (100 μM). Fifteen μg of total protein were loaded in the gels. Transporter O.D. were normalized to β-actin O.D. Uniformity of loading and transfer from gel to PVDF membrane was also controlled through Ponceau S staining. The data (mean ± S.D., n = 3) are expressed as percentage of the normalized transporter expression in control (C) cells. Typical western blot detections are shown at the bottom.

To elucidate if the upregulation of P-gp and MRP2 protein expression by GNT was at transcriptional level, the corresponding mRNA levels were measured after GNT cells exposure (1.0 and 10 μM, 48 h). We found an increase in the mRNA expression of P-gp and MRP2 (+158% and +109% respectively) only at 10 μM of GNT (Fig. 5). As mRNA levels of P-gp and MRP2 remained unchanged after 1.0 μM GNT treatment, we further explored if GNT affected protein synthesis. As seen in Fig. 6, cycloheximide abolished MRP2 and P-gp induction, agreeing well with a regulation at translational level.

We further explored if GNT affected the expression of miR-379, since this miRNA was previously shown to modulate MRP2 protein levels in HepG2 cells. We found that GNT 1.0 μM decreased miR-379 levels (31 ± 10% vs C: 100 ± 34%, p<0.05, n = 6) providing a possible explanation for the occurrence of MRP2 upregulation without changes in mRNA levels.

Regarding P-gp translational regulation, it is known that this transporter is a target of miR-223 in HepG2 cells [12] and that miR-223 is downregulated by GNT in pancreatic cancer cells [46]. We evaluated the effect of GNT (1.0 μM, 48 h) on miR-223 as a potential mediator of P-gp induction and found that its levels were not affected (76 ± 39% for GNT and 100 ± 35% for C, p>0.05, n = 6).

### Involvement of PXR in P-gp and MRP2 induction by GNT

**Fig 7:**
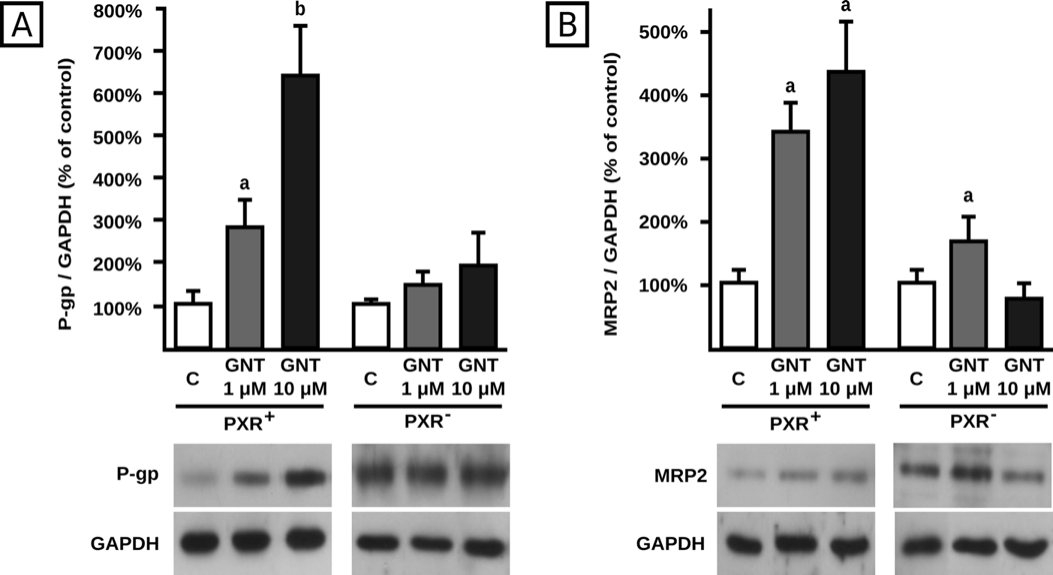
Effect of PXR knock down on transporter induction by GNT. Expression of P-gp (panel A) and MRP2 (panel B) was quantified by western blotting in cell lysates from control siRNA-A and PXR siRNA (h) transfected HepG2 cells (PXR^+^ and PXR^-^ cells, respectively) after 48 h of treatment with GNT (0.1, 1.0, or 10 μM) or vehicle (C). Fifteen μg of total protein were loaded in the gels. Transporter O.D. were normalized to GAPDH O.D. Uniformity of loading and transfer from gel to PVDF membrane was also controlled through Ponceau S staining. The data (mean ± S.D. n = 3) are expressed as percentage of the normalized transporter expression in control (C) cells. Typical western blot detections are shown at the bottom. a: different from control, b: different from GNT 1.0 μM. p<0.05.

PXR is a nuclear receptor involved in the regulation of drug disposition by xenobiotics. It was reported that GNT is capable of activating PXR [47]. Thus, it was of interest to evaluate if the effects of GNT on P-gp and MRP2 were mediated by PXR. We used a siRNA driven mechanism to knock down its expression as previously described and evaluated P-gp and MRP2 protein expression after incubation of these cells with GNT (1.0 and 10 μM, 48 h). The knock down procedure results in decreasing PXR expression by 74%, according to our previous report [37]. As expected, GNT (1.0 μM) increased P-gp and MRP2 expression by 161% and 219% in PXR^+^ (Fig. 7A and B, respectively). The same figures show that GNT induction was completely abolished in PXR^-^ cells for P-gp but not for MRP2. Regarding treatment with GNT at the 10 μM concentration, PXR^+^ cells exhibited increases in protein levels by 543% and 304% for P-gp and MRP2, respectively, consistent with findings in wild-type cells (Fig. 1A and B). Silencing of PXR abolished the induction observed for both transporters.

## Discussion

Hepatocellular carcinoma (HCC) is a high mortality malignancy that represents the fifth leading cancer by incidence worldwide. Chemotherapy is applied when surgical resection, ablation or liver transplantation are not possible and aims to retard the progression of the disease improving life quality and the overall survival of the patients [3]. Multidrug resistance is a common reason of chemotherapy failure. It is characterized by the increase in the expression of one or more ABC transporters such as P-gp, MRP2, MRP3 and BCRP after treatment with their substrates, leading to poorer cytotoxic and/or cytostatic effects [7, 8]. Coadministered drugs or dietary compounds are additional factors that can enhance multidrug resistance by inducing the expression of these transporters [10, 22, 23].

GNT is a phytoestrogen abundant in soy beans. During the last decades it was proposed that GNT ingestion may result in cytoprotection and reduced incidence of specific tumors. GNT beneficial effects may be attributed to its antioxidant properties as activator of the nuclear factor erythroid 2-related factor 2 (Nrf2), to modulation of steroid hormones synthesis, or to activation of signal transduction mechanisms that counterbalance those triggered by endogenous estrogens [27, 48]. *In vitro* evidence suggests an antiproliferative effect of GNT in different cell lines, including HCC models. However, these results were obtained in most of the cases using concentrations above those achieved in the plasma of individuals consuming soy rich diets or supplements containing GNT [27, 30–32]. Additionally, it is uncertain whether GNT affects the efficacy of chemotherapy of HCC. We recently demonstrated that GNT increases the expression of MRP2 and P-gp in human colonic Caco-2 cells, and consequently, reduces the intracellular accumulation of their substrates [49]. Whether GNT induces ABC transporters in cell models resembling HCC, with potential to affect the efficacy of chemotherapy, is unknown. In this work, we investigated the effect of GNT on chemoresistance in a HCC model. We found that GNT, at concentrations reached in plasma of individuals with a high intake of soy or enriched supplements (1.0–10 μM) [31, 32], increased protein expression of P-gp and MRP2 in whole cell lysates in a concentration-dependent manner. Transport activity studies in the presence or absence of specific inhibitors demonstrated that protein synthesis induction was accompanied by increased capability for extrusion of their model substrates. However, no significant differences were seen between activities detected for treatments with GNT at the 1.0 or 10 μM doses. A possible explanation is that newly synthetized P-gp or MRP2 may not be properly localized to the plasma membrane, at least partially. Western blotting of crude plasma membrane showed indeed no substantial difference in MRP2 expression between GNT 1.0 and 10 μM. Conversely, a slight but still significant difference was seen for P-gp expression between both GNT concentrations in the crude membrane preparations, and in consequence other, unknown factors, may explain expression-activity dissociation between GNT 1.0 and 10 μM. These factors could be associated with posttranslational processing of the protein such as glycosylation, phosphorylation, etc., which are known to affect transport activity [10]. These possibilities were not currently explored. Taken together the data suggest that the lowest concentration able to induced protein expression, that is 1.0 μM of GNT, led to maximal transport activity and that a 10-fold increment in concentration no further increased such activity.

There is evidence associating increased P-gp or MRP2 activity with reduced accumulation of chemotherapeutic agents and, consequently, with increased chemoresistance in cancer cells. For instance, Manov et al. [50] reported an induction of P-gp by acetaminophen in HepG2 cells with a concomitant reduction of the accumulation and increased resistance to doxorubicin. An *in vivo* study using a HCC xenograft model also associated P-gp activity with chemoresistance [51]. Regarding the role of MRP2 in HCC chemoresistance, a modulation of its expression was correlated with resistance to vincristine both *in vitro* and in a xenograft model [52]. Based on the current study, we postulate that chemoresistance may be also modulated by GNT.

We further evaluated whether pretreatment with GNT interferes the action of sorafenib (Sfb), the only drug demonstrated to improve overall survival of HCC patients. Our results showed an increase in IC_50_ in GNT pretreated cells either at 1.0 or 10 μM, clearly suggesting decreased Sfb efficacy. The use of selective inhibitors of P-gp and MRP2 (PSC833 and MK571, respectively) confirmed participation of both transporters in chemoresistance. PSC833 also inhibits CYP3A4, which in turn metabolizes Sfb [20, 21, 53]. However, coincubation of Sfb with ketoconazole (CYP3A4 but not P-gp inhibitor) failed to modify chemoresistance mediated by GNT (S1 Table), ruling out a CYP3A4 role in GNT-mediated protection. PSC833 or MK571, when used separately to inhibit either P-gp or MRP2, prevented completely the effect of GNT on Sfb cytotoxicity, as detected in MTT assays. It is known that Sfb undergoes phase I and phase II metabolism leading to the formation of several metabolites which are P-gp or MRP2 substrates and account for Sfb cytotoxicity [20, 21]. Transporter inhibitors also affect Sfb metabolism [53] and, in consequence, the intracellular proportion of the different metabolites is expected to change for each inhibition condition. We speculate that decreased formation of specific metabolites, substrates of the non inhibited transporter, would result in no apparent change in Sfb cytotoxicity due to the induction of this particular transporter.

A P-gp and MRP2 induction by GNT was also observed in Huh7 cell line, which represents another model of HCC [54] (S1 Fig.). This induction was also associated with a protection from Sfb cytotoxicity (S2 Table) showing that the current findings are not restricted to a particular cell line.

The molecular mechanism underlying P-gp and MRP2 induction at the protein level by GNT was first assessed quantifying the mRNA levels of both transporters. For both genes, it was observed an increase in mRNA levels only at the 10 μM concentration, whereas no changes were observed at 1.0 μM, clearly suggesting that different mechanisms are involved. Cycloheximide experiments confirmed that protein synthesis is increased at the 1.0 μM concentration suggesting translational regulation. MicroRNAs have been shown to be important in the modulation of drug transporters. They bind to the mature mRNA inhibiting translation or, alternatively, increasing degradation [44]. P-gp is a known target of miR-223, which in turn was shown to be downregulated by GNT, providing a potential explanation for mediation of P-gp upregulation by GNT [12, 46]. We found no changes in miR-223 expression in response to 1.0 μM GNT treatment, which rules out its participation in our experimental conditions. miR-451, which also modulates P-gp at translational level [55], was additionally evaluated but not detected either in control or in treated cells. Alternative miRNAs, different from miR-223 and miR-451 not currently explored, might be involved. MRP2 was demonstrated to be a target of miR-379 in HepG2 cells [26]. We found that GNT significantly decreased its expression at the 1.0 μM concentration, suggesting a potential participation in translational regulation of MRP2. MicroRNAs are considered novel targets that can influence clinical decision making and eventual therapeutic intervention, including the regulation of key cancer-related pathways such as cell cycle control and the DNA damage response [56]. The fact that miRNA molecules are already entering the clinic as diagnostic and prognostic biomarkers guarantees future studies on their relation to cancer development and therapy. Regulation of expression of miRNAs during chemotherapy of cancer, eventually leading to chemoresistance, should be also a subject of interest in such future studies.

PXR is a nuclear receptor that plays a key role in the regulation of P-gp and MRP2 expression by xenobiotics [22]. It is activated by a wide range of ligands, including GNT [47]. Using a siRNA strategy we demonstrated PXR participation in the transcriptional regulation of both P-gp and MRP2 by 10 μM GNT. It is known that activation of PXR leads to further binding to a distal enhancer region of the P-gp gene bearing direct and everted repeats, thus activating P-gp transcription [22, 25]. A similar mechanism can be proposed for MRP2, as its 5' regulatory region bears an everted repeat at approximately -10kb and an imperfect direct repeat (DR4-like) at approximately -12.5 kb (*in silico* study). We postulate that interaction between GNT and PXR is a necessary event that triggers these transcriptional mechanisms. Interestingly, PXR also mediated the induction of P-gp at the 1.0 μM concentration of GNT. While PXR mediated P-gp regulation at the transcriptional level is well known [22, 25], a translational regulation has not been described yet. Considering that PXR canonical way of action involves the transcriptional regulation of a gene by direct binding to its promoter, our observations might be explained through transcriptional regulation of expression of currently unknown factors, which in turn could regulate translation of drug transporters. Our postulate is supported by recent studies showing that specific cytosolic proteins regulate translation of transporters at ribosomal level [24]. Alternatively, PXR could modulate expression of miRNAs different from those tested currently, also targeting P-gp mRNA, and thus regulating P-gp translation.

In conclusion, we demonstrated P-gp and MRP2 induction by GNT at 1.0 and 10 μM concentrations, at translational and transcriptional levels respectively, with concomitant increase in their transport activities. The nuclear receptor PXR was partially involved in these regulations. Importantly, the effect of GNT resulted in increased resistance to Sfb, the only drug available that improves the overall survival of HCC patients. Although extrapolations to the clinical situation must be cautiously done, the possibility of nutrient-drug interactions leading to enhanced chemoresistance in patients with HCC when consuming a soy-rich diet or GNT supplements needs to be considered.

## Supporting Information

S1 Fig Effect of GNT on ABC transporter expression in Huh7 cells. Huh7 cells were cultured in a medium consisting of DMEM and Ham's F-12 medium (Invitrogen, Carlsbad, CA, USA) at a 1:1 proportion, supplemented with 10% FBS (PAA, Pasching, Austria), 2 mM L-glutamine and a mixture of antibiotics (5 mg/ml penicillin, 5 mg/ml streptomycin). Cells were plated in 6-well plates (350000 cells/well). After 24 h of incubation, cells were exposed to GNT (0.1, 1.0 and 10 μM) or vehicle (C) for 48 h in a treatment medium consisting of the growth medium described above with FBS being replaced by Charcoal-dextran stripped FBS (Hyclone, Logan, UT, USA) Expression of P-gp (panel A), MRP2 (panel B), MRP3 (panel C) and BCRP (panel D) was quantified by western blotting in cell lysates from Huh7 cells as described for HepG2 cells in Materials and Methods. Fifteen μg of total protein were loaded in the gels. Transporter O.D. were normalized to β-actin O.D. Uniformity of loading and transfer from gel to PVDF membrane was also controlled through Ponceau S staining. The data (mean ± S.D. n = 3) are expressed as percentage of the normalized transporter expression in control (C) cells. Typical western blot detections are shown at the bottom. Results showed a significant induction of P-gp (+60%) and MRP2 (+99%) at GNT 10 μM without changes in the expression of MRP3 or BCRP, or at lower GNT concentrations. * different from all the other groups, p<0.05.
(TIF)

S1 Table Effect of ketoconazole coincubation on the protection of sorafenib-induced cytotoxicity by GNT. IC_50_ values (mean ± S.D., n = 4) were obtained through non linear regression. *: different from control (C), p<0.05.
(XLSX)

S2 Table Effect of GNT on Sfb mediated cytotoxicity in Huh7 cells. The effect of P-gp and MRP2 induction by GNT 10 μM (48 h) on Sfb exerted cytotoxicity in Huh7 cells was assessed by the MTT assay. Cells were plated in 96-well plates at a density of 3000 cells/well, cultured for 24 h and exposed to GNT 10 μM or vehicle (C) for 48 h. Then, treatment medium was removed and cells were incubated with Sfb (0–200 μM) for 16 h. Following, MTT assay was performed as described in materials and methods. The use of selective inhibitors of P-gp and MRP2 (PSC833 and MK571, respectively) confirmed participation of both transporters in chemoresistance. IC_50_ values (mean ± S.D., n = 4) showed were obtained through nonlinear regression as described in materials and methods. * different from control (C), a: different from the corresponding group without inhibitor.
(XLSX)
