# Supplementary figures and images for: Regulation of Multidrug Resistance Proteins by Genistein in a Hepatocarcinoma Cell Line: Impact on Sorafenib Cytotoxicity
Source: PLoS One. 2015 Mar 17;10(3):e0119502. doi: 10.1371/journal.pone.0119502 (PMC4364073; doi:10.1371/journal.pone.0119502)

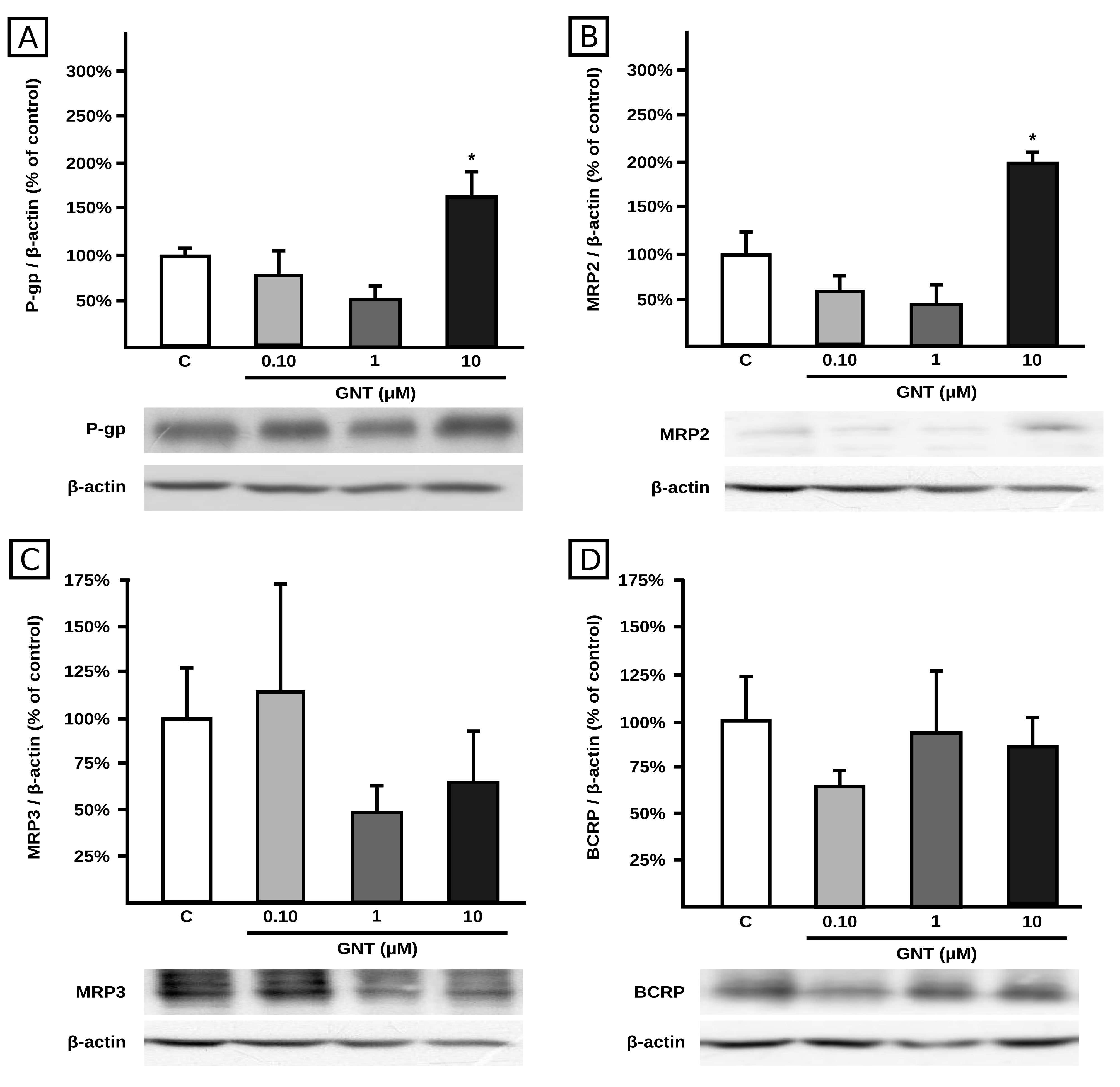

Supplement: S1 Fig — Huh7 cells were cultured in a medium consisting of DMEM and Ham's F-12 medium (Invitrogen, Carlsbad, CA, USA) at a 1:1 proportion, supplemented with 10% FBS (PAA, Pasching, Austria), 2 mM L-glutamine and a mixture of antibiotics (5 mg/ml penicillin, 5 mg/ml streptomycin). Cells were plated in 6-well plates (350000 cells/well). After 24 h of incubation, cells were exposed to GNT (0.1, 1.0 and 10 μM) or vehicle (C) for 48 h in a treatment medium consisting of the growth medium described above with FBS being replaced by Charcoal-dextran stripped FBS (Hyclone, Logan, UT, USA) Expression of P-gp (panel A), MRP2 (panel B), MRP3 (panel C) and BCRP (panel D) was quantified by western blotting in cell lysates from Huh7 cells as described for HepG2 cells in Materials and Methods. Fifteen μg of total protein were loaded in the gels. Transporter O.D. were normalized to β-actin O.D. Uniformity of loading and transfer from gel to PVDF membrane was also controlled through Ponceau S staining. The data (mean ± S.D. n = 3) are expressed as percentage of the normalized transporter expression in control (C) cells. Typical western blot detections are shown at the bottom. Results showed a significant induction of P-gp (+60%) and MRP2 (+99%) at GNT 10 μM without changes in the expression of MRP3 or BCRP, or at lower GNT concentrations. * different from all the other groups, p<0.05. (TIF) [file pone.0119502.s001.tif]
